# Towards Improving the Efficacy of PSMA-Targeting Radionuclide Therapy for Late-Stage Prostate Cancer—Combination Strategies

**DOI:** 10.1007/s11912-023-01458-6

**Published:** 2023-10-20

**Authors:** Daria Arbuznikova, Matthias Eder, Anca-Ligia Grosu, Philipp T. Meyer, Christian Gratzke, Constantinos Zamboglou, Ann-Christin Eder

**Affiliations:** 1 Department of Nuclear Medicine, University Medical Center Freiburg, Faculty of Medicine, University of Freiburg, Hugstetter Str. 55, 79106 Freiburg, Germany; 2 Division of Radiopharmaceutical Development, German Cancer Consortium (DKTK), Partner Site Freiburg, Freiburg, Germany and German Cancer Research Center, Heidelberg, Germany; 3 Department of Radiation Oncology, University Medical Center Freiburg, Faculty of Medicine, University of Freiburg, Freiburg, Germany; 4 Department of Urology, University Medical Center Freiburg, Faculty of Medicine, University of Freiburg, Freiburg, Germany

**Keywords:** Metastatic castration-resistant prostate cancer, Lutetium-177-PSMA-617, Radioligand therapy, Combination therapy, Prostate-specific membrane antigen

## Abstract

**Purpose of Review:**

[^177^Lu]Lu-PSMA-617 is a radiopharmaceutical that emits beta-minus radiation and targets prostate-specific membrane antigen (PSMA)-positive prostate cancer. Despite its clinical success, there are still patients not showing sufficient response rates. This review compiles latest studies aiming at therapy improvement in [^177^Lu]Lu-PSMA-617-naïve and -resistant patients by alternative or combination treatments.

**Recent Findings:**

A variety of agents to combine with [^177^Lu]Lu-PSMA-617 are currently under investigation including alpha radiation-emitting pharmaceuticals, radiosensitizers, taxane chemotherapeutics, androgen receptor pathway inhibitors, immune checkpoint inhibitors, and external beam radiation. Actinium-225 (^225^Ac)-labeled PSMA-targeting inhibitors are the most studied pharmaceuticals for combination therapy or as an alternative for treatment after progression under [^177^Lu]Lu-PSMA-617 therapy.

**Summary:**

Alpha emitters seem to have a potential of achieving a response to PSMA-targeting radionuclide therapy in both initial non-responders or responders to [^177^Lu]Lu-PSMA-617 later developing treatment resistance. Emerging evidence for immunostimulatory effects of radiopharmaceuticals and first prospective studies support the combination of [^177^Lu]Lu-PSMA-617 and immune checkpoint inhibition for late-stage prostate cancer.

## Introduction

The lutetium-177 (^177^Lu)-labeled radiopharmaceutical [^177^Lu]Lu-PSMA-617 (market name Pluvicto™) has been approved by the US Food and Drug Administration (FDA) [1] and European Medicines Agency (EMA) [2] in 2022 as a last-line therapy for metastatic castration-resistant prostate cancer (mCRPC). Patients can be treated with [^177^Lu]Lu-PSMA-617 after progression on anti-hormonal therapy with androgen receptor pathway inhibitors (ARPIs) and taxane chemotherapy. The mode of action of [^177^Lu]Lu-PSMA-617 begins with specific binding of the compound to the prostate-specific membrane antigen (PSMA) [3], a transmembrane protein that is overexpressed in prostate cancer with expression levels rising with higher progression [4, 5]. Upon binding, the pharmaceutical is internalized by PSMA-expressing cells leading to its accumulation and dispersion throughout the cytoplasm [6]. The radiation emitted from ^177^Lu damages the DNA and other macromolecules, ultimately causing cell death. ^177^Lu predominantly emits beta-minus particle radiation which has a relatively low linear energy transfer (LET: amount of energy deposited across a unit of traveled particle track) of 0.2 keV/µm [7]. Still, compared to gamma radiation, beta particles emitted from ^177^Lu have very low tissue penetration ability traveling a mean distance of 0.67 mm [8], thereby damaging only tumor cells in their proximity with limited off-target irradiation of neighboring healthy tissues. The radiation causes single-strand breaks (SSBs) and double-strand breaks (DSBs) in the DNA, either by direct ionization of DNA or indirectly via the formation of highly reactive hydroxyl radicals [9, 10]. These properties render beta-emitting radiopharmaceuticals valuable for the specific targeting of extensively metastasized prostate cancer.

The international, randomized, prospective phase III VISION trial completed the clinical testing phase of [^177^Lu]Lu-PSMA-617, which led to its FDA and EMA approval for mCRPC. The trial assessed the efficacy of the novel treatment comparing [^177^Lu]Lu-PSMA-617 plus standard of care (SOC) and SOC only ([11]; NCT03511664). Permitted SOC included ARPIs (e.g., abiraterone, enzalutamide) and excluded, e.g., chemotherapy and immunotherapy due to unknown risks in combination with [^177^Lu]Lu-PSMA-617. In the radioligand therapy (RLT) arm, patients received a median of five cycles, each at a dose of 7.4 gigabecquerel (GBq) at 6-week intervals. The median overall survival (OS) was significantly better in the RLT arm as compared to SOC only (15.3 months vs*.* 11.3 months). Patients treated with RLT had a median progression-free survival (PFS) of 8.7 months and those treated with SOC 3.4 months. Of note, a substudy of the trial characterized outcomes depending on the patients’ baseline PSMA expression intensity according to positron emission tomography/ computed tomography (PET/CT) imaging. Patients in the highest quartile (whole-body mean standard uptake value (SUV) ≥ 10.2) had a PFS of 14.1 months and those in the lowest quartile (whole body mean SUV < 6.0) 5.8 months. This analysis shows that with low-PSMA expression patients do still benefit from PSMA-targeting RLT with ^177^Lu ([^177^Lu]Lu-PSMA RLT) [12].

The Australian multicenter, randomized, phase II trial “TheraP” ([13]; NCT03392428) enrolled patients previously receiving docetaxel and in most cases ARPIs. The study compared the efficacy of cabazitaxel, one of the standard subsequent treatment regimens, to [^177^Lu]Lu-PSMA-617. RLT was administered every 6 weeks for up to six cycles and cabazitaxel once every 3 weeks for up to ten cycles. The primary endpoint was prostate-specific antigen response (PSA: serum marker for biochemical response) and was defined as the proportion of patients achieving a ≥ 50% PSA decline. The response rates were 66% in the RLT arm and 37% in the cabazitaxel arm.

While [^177^Lu]Lu-PSMA-617 represents a highly specific and effective drug with a favorable safety profile, patients eventually relapse. The OS and PFS are prolonged by 4–5 months when compared to SOC. Some patients do not respond in the first place. Therefore, there is a need for improvements that aim at achieving a response in non-responders or overcome acquired resistance. This review compiles recent studies dealing with rescuing efficacy after failure, promising combination therapies for both [^177^Lu]Lu-PSMA RLT-naïve and -refractory patients, and treatment options after [^177^Lu]Lu-PSMA-617 therapy (Table 1).

**Table 1:** Overview of outcomes from VISION and TheraP and clinical trials combining or sequencing [^177^Lu]Lu-PSMA RLT with other therapies

Treatment	Study	No. of patients; Type of study (pro-/retro-spective)	Prior [^177^Lu]Lu-PSMA RLT status*	PSA decline		Progression-free survival** [months/weeks]		Overall survival [months/weeks]	Adverse events [%]***	
				Any Number *(%)*	≥ 50% Number *(%)*	PSA	Other		3	4
[^177^Lu]Lu-PSMA-617	VISION (Sartor et al. 2021)	831; prospective	-	-	RLT: 177/385 *(46)* SOC: 14/196 *(7)*	-	RLT: 8.7 m SOC: 3.4 m	RLT: 15.3 m SOC: 11.3 m	RLT: 0.8–12.9 SOC: 0.5–4.9	
	TheraP (Hofman et al. 2021)	200; prospective	-	RLT: 81/99 *(82)*^×^ Cab: 54/101 *(53)*^×^	RLT: 65/99 *(66)*^×^ Cab: 37/101 *(37)*^×^	-	RLT: 5.1 m^×^ Cab: 5.1 m^×^	-	RLT: 1–11 Cab: 1–13	
[^225^Ac]Ac-PSMA-617/ [^177^Lu]Lu-PSMA-617 tandem therapy	Khreish et al. 2020	20; retrospective	All refractory Responders Non-responders	18/20 *(90)* - -	13/20 *(65)* 10/12 *(83)* 4/8 *(50)*	- - -	19 w - -	48 w - -	5–10 - -	10 - -
	Rosar et al. 2021, *Theranostics*	17; retrospective	Refractory	10/17 *(59)*	5/17 *(29)*	3.7 m	-	*PR vs. SD/PD by imaging:* not reached vs. 8.3 m	6	0
	Kulkarni et al. 2019	23	Refractory	13/23 *(57)*	10/23 *(43)*	-	21 w	33 w	9	0
	Rosar et al. 2021, *Pharmaceutics*	15; retrospective	Naïve	14/15 *(93)*	8/15 *(53)*	9.1 m	-	14.8 m	13	0
[^225^Ac]Ac-PSMA-RLT^□^ after [^177^Lu]Lu-PSMA-RLT^□^	Feuerecker et al. 2021	26; retrospective	All refractory Responders Non-responders	23/26 *(88)* 16/18 *(89)* 7/8 *(88)*	17/26 *(65)* 13/18 *(72)* 4/8 *(50)*	3.5 m - -	4.1 m - -	7.7 m - -	12–31 - -	0–8 - -
	Sathekge et al. 2020	73; retrospective	All Naïve Refractory	- - -	- - -	- - -	15.2 m 16.5 m 5.1 m	18 m - -	1–7 - -	3 - -
	Yadav et al. 2020	28; prospective	All Naïve Refractory	22/28 *(79)* - -	11/28 *(39)* 7/13 *(54)* 4/15 *(27)*	- - -	12 m 12 m 10 m	17 m 17 m 16 m	4 - -	0 - -
	Zacherl et al. 2021	14; retrospective	All Naïve Refractory	11/14 *(79)* 3/3 *(100)* 8/11 *(73)*	7/14 *(50)* 2/3 *(67)* 5/11 *(45)*	- - -	- - -	- - -	7–21 - -	0 - -
[^177^Lu]Lu-PSMA RLT^□^ and radiosensitizers	Pathmanandavel et al. 2022 **Idronoxil**	56; prospective	Naïve	48/56 *(86)*	34/56 *(61)*	7.5 m	-	19.7 m	7	0
[^177^Lu]Lu-PSMA-617 and ARPIs	Rosar et al. 2022 **Enzalutamide**	10; retrospective	Refractory	10/10 *(100) (best PSA response)* 6/10 *(60) (after 2*^*nd*^* cycle)*	7/10 *(70)* *(best PSA response)* 5/10 *(50)* *(after 2*^*nd*^* cycle)*	-	-	12.6 m	10	0
	Suman et al. 2021 **Abiraterone**	58; retrospective	Naïve	Monotherapy 22/38 *(58)*	8/38 *(21)*	-	7 m	8 m	0	0
				Combination 16/20 *(80)*	3/20 *(15)*	-	Not reached	16 m	0	0
[^177^Lu]Lu-PSMA-RLT^□^ and immunotherapy	Sandhu et al. 2022 **Pembrolizumab**	37; prospective	Naïve	-	28/37 *(76)*	8.2 m	11.2 m	17.8 m	G2/3 anemia: 8 G3 immune-related: 27	
	Aggarwal et al. 2021 **Pembrolizumab**	18; prospective	Naïve	-	5/18 *(28)*	-	6.5 m	-	6	

^*^Responders = initial responders subsequently developing resistance; non-responders = no benefit from the start

^**^Progression-free survival = based on PSA levels (= PSA), or imaging, symptoms, PSA either as single parameter or combinations (= other)

^***^Minimum and maximum rates across various categories of effects

^**□**^When “RLT” was written, it was not specified whether PSMA-617 or PSMA-I&T were used as ligands

^×^By intention to treat (prior to patient withdrawal)

*Cab* cabazitaxel, *RLT* radioligand therapy, *PSA* prostate-specific antigen, *SOC* standard of care, *PR* partial response, *SD* stable disease, *PD* progressive disease, *3* grade 3 adverse events, *4* grade 4 adverse events

## Alpha Therapy

Targeted radionuclide therapy with alpha particle emitters might have advantages over therapy with beta emitters under certain circumstances. Alpha emitters have a higher LET of around 80 keV/µm and shorter particle range of up to 100 µm as compared to beta emitters [10]. These properties are associated with induction of a higher number and longer persistence of DNA DSBs and more pronounced cytotoxicity [9, 14]. The highly damaging radiation not only affects tumors but also the physiologically PSMA-expressing salivary glands limiting the applicable dose for alpha therapy [15]. Nevertheless, targeted alpha therapy represents an attractive option for patients that develop resistance to the beta-emitting [^177^Lu]Lu-PSMA RLT or are not responsive in the first place.

Several (mostly retrospective) studies assessed the outcomes of sequencing PSMA-targeting RLT with actinium-225 (^225^Ac) after failure of [^177^Lu]Lu-PSMA RLT or of an administration of both in tandem. Unfortunately, not all studies provided information on the quality of the patients’ responses to prior [^177^Lu]Lu-PSMA RLT, the knowledge of which would have been helpful in rating the study outcomes. Three studies [16, 19, 20^177^Lu]Lu-PSMA RLT but then developed resistance and those that did not respond from the beginning. The responses were rated based on changes in PSA levels. However, the criteria for an insufficient response, that justified the patients’ eligibility for alpha or tandem therapy, were somewhat different across studies. Therefore, the available information is given for each study presented in the following paragraphs.

### Sequencing Actinium After Lutetium-Labeled RLT

A retrospective study analyzed the therapeutic and adverse effects of [^225^Ac]Ac-PSMA-I&T in 14 mCRPC patients having a history of prior [^177^Lu]Lu-PSMA RLT in 11/14 cases (79%). Four of those patients experienced treatment failure or early progression after a median of two cycles. Seven patients exhibited an initial response followed by later progression after a median of four cycles. For subsequent alpha therapy, patients received a median of two cycles [^225^Ac]Ac-PSMA-I&T at a dose of 100 kBq/kg. After the last cycle, a ≥ 50% PSA decline was reported for 7/14 (50%) patients. Individuals that previously underwent [^177^Lu]Lu-PSMA RLT, had a slightly worse response (5/11 (45%)) [16••].

It has to be highlighted that patients already with a history of progression on [^177^Lu]Lu-PSMA RLT show an inferior outcome after [^225^Ac]Ac-PSMA RLT as compared to RLT-naïve patients [17•]. This is in line with the more general observation that in patients with metastatic prostate cancer, sequencing of treatment options with similar mode of action (MoA) leads to inferior clinical benefits when compared to a sequence of different treatment modalities with different MoAs. A prospective study supported this observation [18••]: The efficacy of [^225^Ac]Ac-PSMA-617 was compared in 15 [^177^Lu]Lu-PSMA-617-refractory and 13 [^177^Lu]Lu-PSMA-617-naïve patients. For the refractory group, the authors provided no information regarding the quality of the response to prior [^177^Lu]Lu-PSMA RLT. Targeted alpha therapy was administered at a dose of 100 kBq/kg in 8-week intervals; the median number of cycles was three. The PSA outcome diverged between the two subgroups: 4/15 (26.6%) [^177^Lu]Lu-PSMA-617-refractory patients achieved a PSA decline > 50% as opposed to 7/13 (53.8%) in the [^177^Lu]Lu-PSMA-617-naïve group. However, concerning median PFS and OS, the two groups did not significantly differ (10 vs. 12 months and 16 vs. 17 months, respectively).

Feuerecker et al. [19••] retrospectively analyzed the response to [^225^Ac]Ac-PSMA-617 in 26 heavily pretreated patients all refractory to prior [^177^Lu]Lu-PSMA RLT. The patients were subcategorized according to their previous response to RLT, which was either initial treatment failure or loss of response upon rechallenge. 19/26 (73%) patients responded to [^177^Lu]Lu-PSMA RLT which was determined according to any PSA decline. A median of two cycles of [^225^Ac]Ac-PSMA RLT was given at an 8-week interval. 17/26 (65%) patients achieved a ≥ 50% PSA decline. PSA-PFS and OS were reported to be 3.5 and 7.7 months, respectively. Previous [^177^Lu]Lu-PSMA RLT non-responders were less likely to show a 50% PSA decline than initial responders (with 4/8 (50%) vs. 13/18 cases (72%)).

### Actinium – and Lutetium-Labeled RLT In Tandem

Besides sequencing [^225^Ac]Ac-PSMA-targeting inhibitors after [^177^Lu]Lu-PSMA-targeting inhibitors, an emerging approach is the simultaneous administration of alpha and beta emitters. Referred to as [^225^Ac]Ac-PSMA/[^177^Lu]Lu-PSMA tandem therapy or [^225^Ac]Ac-PSMA augmented [^177^Lu]Lu-PSMA RLT, the combination treatment might have several advantages: Radionuclides with different particle ranges and LETs might act synergistically in targeting metastases of diverse size, with heterogeneous PSMA expression and acquired radiation resistance. Furthermore, applying both emitters in combination allows for dose reduction of either, thereby potentially lowering salivary gland toxicity [20••].

Several publications reported the administration of [^225^Ac]Ac-PSMA/[^177^Lu]Lu-PSMA tandem therapy in heavily pretreated patients with mCRPC that previously underwent [^177^Lu]Lu-PSMA RLT. In one retrospective analysis, the patients were subcategorized into those who initially responded but developed resistance to [^177^Lu]Lu-PSMA RLT or who were initially refractory. An eventual “insufficient response” in both groups was defined as a PSA increase or < 50% decrease after which the patients received the tandem therapy. The experimental treatment was a single course of [^225^Ac]Ac-PSMA-617 (median: 5.3 MBq) followed by [^177^Lu]Lu-PSMA-617 (median: 6.9 GBq) administered mostly on two consecutive days. In case of PSA decline or stabilization, patients were given [^177^Lu]Lu-PSMA-617 as maintenance therapy. During follow-up, a PSA decline ≥ 50% was recorded in 13/20 (65%) patients. The median PFS and median OS after tandem therapy were 19 weeks and 48 weeks, respectively. When patients were grouped according to their response history to [^177^Lu]Lu-PSMA-targeting monotherapy, no significant differences in outcome were reported. Importantly, the opportunity of lowering the dose of the alpha emitter in the tandem approach prevented severe adverse effects on salivary glands [20••].

A different cohort [21••] also received a single cycle of [^225^Ac]Ac-PSMA-617/ [^177^Lu]Lu-PSMA-617 tandem therapy. After the combined treatment, a partial response (> 50% PSA decrease) was reported for 5/17 (29.4%) patients. The median PSA-PFS for all patients was 3.7 months. The median OS was not reached by patients with partial remission and amounted to 8.3 months in those with stable or progressive disease. The outcomes in this cohort were worse than in Khreish et al. [20••], probably due to the more challenging cohort of patients. Here [21••], patients with documented progression according to both, PSA and PSMA PET/CT, were included. Progression was defined as both a > 25% PSA increase relative to the previous cycle and an emergence of new lesions or greater uptake in lesions on PET/CT. In contrast, Khreish et al. [20••] evaluated insufficient responders based on a PSA increase or a decrease that was < 50%.

Additionally, Kulkarni et al. [22] reported a rescued response after one cycle of [^225^Ac]Ac-PSMA-617/ [^177^Lu]Lu-PSMA-617 tandem therapy in a group of 23 mCRPC patients refractory to [^177^Lu]Lu-PSMA RLT. A > 50% PSA decline was noted in 10/23 (43%) patients. The PFS and OS were reported to be 21 and 33 weeks, respectively.

Interestingly, across the three abovementioned reports including [^177^Lu]Lu-PSMA RLT-refractory patients only, the average PSA response rate after tandem therapy (46%, range 29–65%) was the same as in the VISION trial (46%) and severe adverse events were not occurring more frequently than in the VISION or TheraP trials (Table 1**)**, although inclusion criteria were very different. Only one publication [20••] makes a distinction between initial responders and non-responders to prior [^177^Lu]Lu-PSMA RLT in the evaluation of the outcome. Given the good results, [^225^Ac]Ac-PSMA/[^177^Lu]Lu-PSMA tandem therapy could be more frequently considered in the future to treat late-stage, [^177^Lu]Lu-PSMA RLT-refractory mCRPC.

Lastly, one retrospective study assessed the outcome of [^225^Ac]Ac-PSMA-617/ [^177^Lu]Lu-PSMA-617 tandem therapy in 15 [^177^Lu]Lu-PSMA RLT-naïve patients having a poor prognosis [23]. After treatment with two cycles [^177^Lu]Lu-PSMA-617 and at least one [^225^Ac]Ac-PSMA-617 augmentation, the authors reported a ≥ 50% PSA decline in 8/15 (53%) patients. The PFS and OS amounted to 9.1 and 14.8 months, respectively. Remarkably, the PSA response is similar (53% vs. 57%) to the outcome in mCRPC patients with more favorable disease characteristics treated with two cycles of [^177^Lu]Lu-PSMA-617 monotherapy [24], but the number of patients is small.

## Radiosensitizers

When ionizing radiation as monotherapy lacks efficacy, radiosensitizers help to overcome this limitation, ideally, by lowering the threshold for toxicity in cancer cells but not in normal cells. For [^177^Lu]Lu-PSMA RLT of mCRPC, radiosensitizers facilitating apoptosis, inhibiting mitosis, and inducing DNA damage are currently under clinical investigation. However, there is hardly any data on the combined application in the [^177^Lu]Lu-PSMA-617-refractory setting. Larger studies address the benefits of such combinations in [^177^Lu]Lu-PSMA RLT-naïve patients.

The prospective single-center phase I/II LuPIN trial investigated 56 mCRPC patients that were allocated into three groups, each receiving different doses of the radiosensitizer idronoxil together with [^177^Lu]Lu-PSMA-617 [40•]. Idronoxil is a derivative of the isoflavone genistein, a compound isolated from soy with anticancer properties. It was found to be cytotoxic to prostate cancer cells triggering apoptosis in vitro [41]. Compared to patients treated with [^177^Lu]Lu-PSMA-617 in the VISION trial, patients in the LuPIN trial exhibited a better 50% PSA decline rate (61% vs. 46%) and a better median OS (19.7 months vs. 15.3 months). Adverse effects were mainly of grade 1 and the different doses of idronoxil did not produce significantly different outcomes or toxicity.

A combination of [^177^Lu]Lu-PSMA-617 with taxane chemotherapy is also under evaluation. A case study reported the successful use of low-dose docetaxel and [^177^Lu]Lu-PSMA RLT in a mCRPC patient [42]. In the mCRPC setting, treatment with docetaxel and beta-emitting RLT was also analyzed in a phase I study that enrolled 15 patients evaluating the PSMA-targeting antibody [^177^Lu]Lu-J591 ([43]; NCT00916123). No dose-limiting toxicity was identified. Recently, the LuCAB trial was launched to assess the safety and efficacy of cabazitaxel in addition to [^177^Lu]Lu-PSMA-617 in heavily pretreated mCRPC (NCT05340374).

Inhibitors of the enzyme poly(ADP-ribose) polymerase (PARP) are valuable drugs targeting the DNA damage response (DDR) for the treatment of advanced prostate cancer. In particular, phase III studies on the use of PARP inhibitors have been published in mCRPC patients as mono- and combination therapies using olaparib, niraparib, talazoparib, and rucaparib. As of today, only olaparib is approved as mono- and combination therapy with abiraterone by the EMA, and olaparib and rucaparib are approved by the FDA [44–46]. PARP is involved in the early phase of DNA SSB repair, specifically in damage site recognition and protection of SSBs from cellular nucleases [47, 48]. With PARP inhibited, SSBs can transform to much more toxic DSBs upon initiation of replication. Since ^177^Lu-labeled RLT induces SSBs, combining RLT with PARP inhibitors might achieve a synergistic effect on tumor control, especially in DNA repair-deficient prostate cancer. However, data on efficacy of combining the two treatments for prostate cancer are very limited. On preclinical level, PARP inhibitors (olaparib, talazoparib) did not add to the DNA-damaging effect of [^177^Lu]Lu-PSMA RLT in vitro. Only veliparib augmented the amount of DNA DSBs [49]. In an in vivo therapy study on PC-3 PIP xenograft-bearing mice, the combination of PARP inhibitors and [^177^Lu]Lu-PSMA RLT did not improve tumor control [49]. The study sequenced [^177^Lu]Lu-PSMA RLT first, followed by PARP inhibitors. It is possible that a reverse treatment sequence that weakens the DNA SSB repair capacity prior to [^177^Lu]Lu-PSMA RLT might have led to a different outcome. On the clinical level, a phase I study combining [^177^Lu]Lu-PSMA-617 and olaparib (LuPARP, NCT03874884) for genetically unselected mCRPC is being conducted and is estimated to be completed by December 2023.

Curiously, DNA-damaging pharmaceuticals are being investigated for their potential to modulate PSMA expression levels. High PSMA expression is associated with mutations in DDR genes such as *BRCA2* or *ATM* [5] suggesting a role of PSMA in DNA damage repair. Remarkably, a recent work [39] identified the topoisomerase-2 inhibitors daunorubicin and mitoxantrone as effective inducers of PSMA protein expression in prostate cancer cell cultures and a patient-derived xenograft. These effects, in turn, might potentiate PSMA RLT efficacy.

## Androgen Blockade

The efficacy of [^177^Lu]Lu-PSMA RLT of mCRPC is influenced by the extent of PSMA expression of tumors. Patients with low PSMA expression in mCRPC are less likely to experience a PSA response after PSMA RLT [25]. In case of low PSMA expression, PFS and OS are still extended more than with SOC [12] but outcomes for these patients might be improved with measures aiming at increasing PSMA levels. PSMA expression is suppressed by androgens and rises upon androgen deprivation [26, 27]. Furthermore, there is evidence suggesting that androgen blockade with ARPIs leads to a PSMA upregulation in mCRPC, specifically [30, 31], which might allow a synergistic tumor control in combination with PSMA RLT. However, evidence of an improved RLT efficacy following pretreatment with ARPIs is lacking so far.

### Modulation of PSMA Levels

In vitro and in vivo studies on prostate cancer cell lines and tumor xenografts with varying basal PSMA expression levels and androgen dependencies showed an upregulation of PSMA by 1.5- to 25-fold following exposure to enzalutamide [28–30]. The effects occurred after treatment for 1 to 4 weeks. PSMA upregulation after enzalutamide pretreatment did, however, not enhance efficacy of [^177^Lu]Lu-PSMA-617 in a preclinical CRPC mouse model [29].

In a patient with low PSMA-expressing mCRPC in [^68^Ga]Ga-PSMA-11 PET imaging, a 5-month treatment with enzalutamide enhanced [^68^Ga]Ga-PSMA-11 uptake which rendered him eligible for [^177^Lu]Lu-PSMA-617 therapy [30]. After two cycles, the patient exhibited a good response. Furthermore, in a small prospective cohort of mCRPC patients, abiraterone or enzalutamide administration induced an increase of the SUV on [^68^Ga]Ga-PSMA PET imaging by day nine which was partly sustained until day 28 [31]. This occurred simultaneously with diverse PSA outcomes and an increase in total tumor volume. The significance of the PET data is debatable as changes in tumor volume influence the results. The maximum SUV decreased for hormone-sensitive disease as early as day nine after starting androgen deprivation therapy simultaneously with a drop in PSA levels and total tumor volume [31]. Additionally, since many mCRPC patients are already under treatment with ARPIs when they start RLT, possible benefits might be experienced by a limited number of patients. On the other hand, it seems as if patients that suspended treatment with enzalutamide due to failure, still show increased PSMA levels upon short-term retreatment [32, 33]. Larger, prospective trials may be performed to confirm the potential of ARPIs to enhance PSMA levels in mCRPC. Such an effect might be valuable for [^177^Lu]Lu-PSMA RLT of low-PSMA mCRPC.

### Combined Androgen Blockade and Lutetium-Labeled RLT

Limited evidence points to a benefit of combined ARPI and [^177^Lu]Lu-PSMA RLT. A very recent retrospective study demonstrated a rescuing effect of adding enzalutamide on response to [^177^Lu]Lu-PSMA-617 in patients that were about to become resistant to RLT [34••]. Ten patients with mCRPC were administered [^177^Lu]Lu-PSMA RLT for a median of two cycles before imminent treatment failure according to the PSA response was anticipated. At this point, the patients started concurrent enzalutamide with a median of additional five cycles of [^177^Lu]Lu-PSMA RLT. 5/10 patients exhibited a PSA decrease > 50% after the second enzalutamide augmented RLT cycle. 7/10 patients experienced a 50% PSA decrease at any point after receiving the combined treatment. However, patients with a history of enzalutamide failure had less benefit from the combined treatment as compared to enzalutamide-naïve patients (PSA response in 2/4 vs. 5/6, respectively).

The Australian randomized phase II trial ENZA-p is investigating a combined administration of [^177^Lu]Lu-PSMA-617 and enzalutamide in patients with mCRPC that have not had RLT before (NCT04419402; [35]). The study is designed to evaluate the potential of overcoming resistance towards enzalutamide by adding RLT.

Besides enzalutamide, the outcomes of a retrospective study of combined [^177^Lu]Lu-PSMA RLT and abiraterone were published by Suman et al. [36•]. Twenty patients with mCRPC were given 1000 mg abiraterone daily combined with an average of three [^177^Lu]Lu-PSMA-617 therapy cycles and compared to 38 patients receiving [^177^Lu]Lu-PSMA RLT monotherapy. Only 21% of patients receiving monotherapy and 15% receiving combined treatment showed a PSA decrease > 50%. However, the median PFS was seven months in the monotherapy group and was not reached in the combination group at a median follow-up of ten months.

## Immunotherapy

Radiation-mediated cell death can induce tumor immunogenicity due to release of cell debris with tumor antigens and their uptake by antigen presenting cells and presentation to T cells [7]. Furthermore, sublethal irradiation activates the DNA sensing cGAS-STING pathway, which induces immunostimulatory type I interferon production. These radiation-mediated effects might enhance the efficacy of immunotherapy with, e.g., immune checkpoint inhibitors. Direct immunomodulatory effects of focal irradiation with external beam radiotherapy are well described. Emerging preclinical evidence suggests that RLT can also alter the tumor microenvironment and that the combination of RLT and immune checkpoint inhibition has a synergistic inhibitory effect on cancer growth [50, 51].

Czernin et al. [52] showed in a preclinical xenograft mouse model for CRPC that combining [^225^Ac]Ac-PSMA-617 and an anti-programmed death 1 (PD-1) antibody provides an advantage for tumor growth control and survival. As an alternative strategy to boost an antitumor immune response, [^225^Ac]Ac-PSMA-617 was administered to the same mouse model together with an agonist of the STING system [53]. The combination treatment completely suppressed tumor growth. The authors even reported a persisting immune memory upon rechallenge of the mice with the cancer cells.

Whether [^177^Lu]Lu-PSMA RLT can act synergistically with immunotherapy needs to be clarified. Several clinical trials are registered. A phase Ib trial evaluated the safety and potential of a single [^177^Lu]Lu-PSMA-617 dose to induce immune priming in order to increase efficacy of pembrolizumab in 18 patients with chemo-naïve mCRPC ([54]; NCT03805594). For the trial, patients were subjected to various sequencing strategies of [^177^Lu]Lu-PSMA-617 and pembrolizumab. A complete or partial response was achieved in 8/18 (44%) patients. Radiographic PFS was 6.5 months and a rather low 50% PSA decline rate of 28% was reached. Another phase I/II trial (NCT03658447, PRINCE) evaluated the combined administration of up to six cycles of [^177^Lu]Lu-PSMA-617, 6-weekly, and 3-weekly pembrolizumab for up to 35 cycles in 37 mCRPC patients, most of whom have had prior chemotherapy. A 50% PSA decrease was found in 28/37 (76%) patients and an imaging-based partial response in 7/10 (70%). The median radiographic PFS was 11.2 months, respectively [55•]. Of note, the combination treatment was more effective in this small study than the pembrolizumab monotherapy evaluated by the phase II KEYNOTE-199 trial that also included mCRPC patients with a history of chemotherapy (NCT02787005). An objective response rate of 5%, a radiographic PFS of 2.1 months and a 50% PSA decline rate of 7% were reported for patients with measurable disease per RECIST v1.1 after treatment with the monotherapy [56]. A more recently initiated randomized trial tests the combination of ipilimumab and nivolumab plus [^177^Lu]Lu-PSMA-617 compared to [^177^Lu]Lu-PSMA-617 monotherapy (EVOLUTION, NCT05150236). Unfortunately, so far, the use of pembrolizumab in prostate cancer has led to disappointing results in a series of phase III studies in metastatic hormone-sensitive prostate cancer and different stages of mCRPC. Four large randomized controlled trials combining pembrolizumab with chemotherapy, PARP inhibitors and novel second-generation hormonal agents were closed early for futility.

## External Beam Radiation Therapy

Combining RLT with ionizing radiation from an external source may provide a benefit with regard to escalation of the radiation dose to the tumor. Since the two sources of radiation have differing organs at risk, their simultaneous application can increase damage to tumor tissue while sparing healthy tissues. A preclinical study in mice bearing androgen-independent PC-3 PIP xenografts found an additive effect of the combination of proton beam radiotherapy and [^177^Lu]Lu-PSMA-617 on tumor control [37].

Interestingly, it was shown that exposure to fractionated external beam irradiation increased the protein levels of PSMA in prostate cancer cell cultures and a patient-derived xenograft [38]. Cell lines were both androgen-dependent and -independent and the xenograft was mCRPC-derived. Six fractions of 2 Gray induced PSMA protein expression by approximately two-fold to ten-fold. If such effects can be reproduced in patients, irradiating tumors prior to administration of [^177^Lu]Lu-PSMA RLT might enhance the uptake and internalization of the radiopharmaceutical.

Clinical data on the combination of external irradiation and [^177^Lu]Lu-PSMA-targeting inhibitors is, so far, very limited. Two case reports exist describing the combined application in heavily pretreated mCRPC patients with brain metastasis [39]. Considering the multitude of tumors in late-stage prostate cancer, the feasibility of external irradiation might be limited to only larger tumors.

## Conclusions

**Fig. 1:**
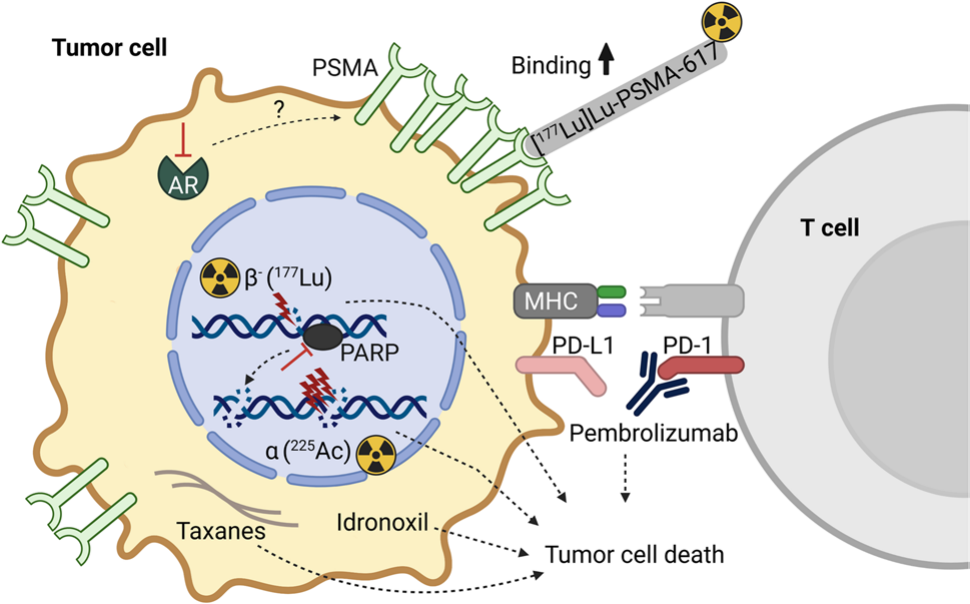
Modes of action of therapeutic agents for late-stage prostate cancer that are under evaluation for a combination therapy with [^177^Lu]Lu-PSMA-617. [^225^Ac]Ac-PSMA RLT is one of the most promising options for mCRPC patients that experienced [^177^Lu]Lu-PSMA RLT failure. Alpha particles exhibit a higher LET than *β*^−^ particles, thus, causing denser DNA damage in the form of DSBs. For [^177^Lu]Lu-PSMA RLT-naïve patients, immunotherapy with pembrolizumab emerges as a therapeutic modality with high potential in the combination with the radiopharmaceutical. Pembrolizumab binds to PD-1 receptors on T cells and abrogates the immunosuppressive interaction between immune checkpoint proteins on T cells and tumor cells. This leads to an immune attack on the tumor cells in response to recognition of neoantigens presented on major histocompatibility complexes (MHC). The genistein-derivative idronoxil facilitates cell death and led to a longer OS in combination with [^177^Lu]Lu-PSMA-617 as compared to the monotherapy with the latter. Other potentially radiosensitizing candidates are the taxane chemotherapeutic cabazitaxel and PARP inhibitor olaparib that are investigated in clinical trials. Taxanes interfere with microtubule dynamics, thereby, preventing the normal process of mitosis. PARP inhibitors suppress the sensing and repair of DNA SSBs which leads to the formation of DSBs. It remains to be investigated whether ARPI-induced PSMA upregulation, and thus increased binding capacity for [^177^Lu]Lu-PSMA-617, can translate into a higher efficacy of the radiopharmaceutical therapy. Another question is whether patients that are already receiving ARPIs can benefit from the approach. *Created with BioRender.com*

[^177^Lu]Lu-PSMA RLT has recently been approved as a last-line therapy for mCRPC after failure of ARPIs and chemotherapy with taxanes. Treatment options for non-responders and patients with acquired resistance are, hence, limited. For the post-[^177^Lu]Lu-PSMA RLT setting, [^225^Ac]Ac-PSMA-617/ [^177^Lu]Lu-PSMA-617 tandem therapy represents a promising alternative eventually restoring response. Administration of targeted alpha monotherapy can also rescue the efficacy of RLT in patients refractory to treatment with beta-emitting radioligands. It is important to highlight that half of initial [^177^Lu]Lu-PSMA RLT non-responders showed improvements with [^225^Ac]Ac-PSMA RLT but the studies that separately report outcomes according to the prior response history are very few, of small size and retrospective. A new, intriguing option for improving [^177^Lu]Lu-PSMA RLT in patients naïve to the radiopharmaceuticals might be the combination with the immune checkpoint inhibitor pembrolizumab with its potential for synergistically remodeling the tumor microenvironment. Whether addition of ARPIs to upregulate PSMA expression, specifically enzalutamide, is able to enhance [^177^Lu]Lu-PSMA RLT efficacy, needs to be clarified. Since many mCRPC patients are already on continued ARPIs when commencing [^177^Lu]Lu-PSMA RLT, the number of patients that could potentially benefit from this strategy is limited. The options for therapeutics to combine with [^177^Lu]Lu-PSMA-617 that were investigated or are currently under evaluation in clinical studies are summarized in Fig. 1.
